# *Wolbachia* limits pathogen infections through induction of host innate immune responses

**DOI:** 10.1371/journal.pone.0226736

**Published:** 2020-02-20

**Authors:** Donghui Zhang, Yingfan Wang, Kun He, Qinggui Yang, Maoqing Gong, Minjun Ji, Lin Chen

**Affiliations:** 1 School of International Education, Nanjing Medical University, Nanjing, Jiangsu, China; 2 Jiangsu Province Key Laboratory of Modern Pathogen Biology, Nanjing Medical University, Nanjing, Jiangsu, China; 3 Program of “5+3” Integrative Clinical Medicine, School of Basic Medical Sciences, Nanjing Medical University, Nanjing, Jiangsu, China; 4 Program of Medical Imaging, School of Medical Imaging, Nanjing Medical University, Nanjing, Jiangsu, China; 5 Jiangsu International Travel Healthcare Center, Nanjing, Jiangsu, China; 6 Shandong Academy of Medical Sciences, Shandong Institute of Parasitic Disease Prevention and Control, Jining, Shandong, China; 7 Department of Pathogen Biology, Nanjing Medical University, Nanjing, Jiangsu, China; 8 Key Laboratory of Infectious Diseases, School of Public Health, Nanjing Medical University, Nanjing, Jiangsu, China; University of Zurich, SWITZERLAND

## Abstract

**Background:**

*Wolbachia* has been reported to suppress a variety of pathogen infections in mosquitoes, but the mechanism is undefined. Two possibilities have been proposed. One is that *Wolbachia* activates host immune responses, and the other one is that *Wolbachia* competes with pathogens for limited nutrients.

**Methodology/Principal findings:**

In this study, we compared host immune responses and the densities of two different strains of *Wolbachia* in naturally occurring parental and artificially created hybrid host genetic backgrounds. No significant difference in *Wolbachia* density was found between these hosts. We found that *Wolbachia* could activate host innate immune responses when the host genetic profile was different from that of its natural host. When these hosts were challenged with pathogenic bacteria, mosquitoes in new host-*Wolbachia* symbioses had a higher survival rate than in old host-*Wolbachia* symbioses.

**Conclusions/Significance:**

The presence of *Wolbachia* per se does not necessarily affect pathogen infections, suggesting that a competition for limited nutrients is not the main reason for *Wolbachia*-mediated pathogen suppression. Instead, host immune responses are responsible for it. The elucidation of an immunity nature of PI is important to guide future practice: *Wolbachia* may be genetically engineered to be more immunogenic, it is desired to search and isolate more strains of *Wolbachia*, and test more host-*Wolbachia* symbioses for future applications. Our results also suggest *Wolbachia*-based PI may be applied to naturally *Wolbachia*-infected mosquito populations, and extend to the control of a broader range of mosquito-borne diseases.

## Introduction

Mosquito-borne diseases are one of the major public health problems. With increasing globalization, urbanization and global warming, the threat of mosquito-borne diseases is growing. Traditional and emerging mosquito-borne diseases, such as malaria, dengue, West Nile fever, Japanese encephalitis, chikungunya fever and Zika, have seriously affected human health and economic development [1, 2]. However, lack of effective vaccines and specific drugs for mosquito-borne diseases (such as dengue), as well as the development of resistance to therapeutic drugs in some pathogens (such as malaria), have contributed to this situation. Therefore, one of the main measures for the prevention and control of mosquito-borne diseases is still mosquito control. Chemical control has been the main method in mosquito control programs. However, continuous and large-scale insecticide usage has led to the emergence and development of resistance in mosquito vectors [3], and the negative effects of insecticides on human health and the environment should not be ignored [4, 5]. Recently, several biological approaches were called upon for the control of mosquito populations, including the introduction of *Wolbachia* [6–8].

The endosymbiotic bacterium *Wolbachia* is maternally inherited, infecting >65% of all insect species and ~28% of the surveyed mosquito species [9, 10]. *Wolbachia* can regulate the host's reproductive processes. For example, cytoplasmic incompatibility (CI) interferes with the normal development of a zygote formed by a sperm of *Wolbachia*-infected mosquito and egg of an uninfected or an incompatible strain of *Wolbachia*-infected mosquito [11]. CI provides a reproductive advantage to infected females over uninfected females, resulting in the invasion of *Wolbachia* into a population. *Wolbachia* can also inhibit pathogen infection of the host via pathogen interference (PI) phenomenon[12]. Studies have shown that *Aedes aegypti* mosquitoes artificially infected with *Wolbachia* have increased resistance to dengue virus, Zika virus, chikungunya virus, yellow fever virus, *Plasmodium gallinaceum*, filaria and certain bacteria [7, 13–16]. After transient somatic infections of *Wolbachia*, *Anopheles gambiae* has significantly reduced infection intensity of *Plasmodium berghei* [17]. Bian *et al*. established a stable *Wolbachia* infection in *Anopheles stephensi* which conferred resistance in the mosquito to *Plasmodium falciparum* [18]. Micieli *et al*. reported that *Wolbachia* infection of *Cx*. *quinquefasciatus* laboratory strain increased host resistance to West Nile virues infection [19].

Currently, *Wolbachia*-infected *Ae*. *Aegypti* mosquitoes have been released in dengue-endemic area as a population replacement strategy. For example, in northern Australia and central Vietnam such mosquitoes were released to replace the local *Wolbachia*-negative *Ae*. *aegypti* population and reduce dengue virues(DENV)-transmission capacity [20]. A mathematical model predicts that establishment of *w*MelPop-infected *Ae*. *aegypti* at high frequency in a dengue endemic setting would result in complete abatement of DENV [21]. However, the long-term effects of artificial release of *Wolbachia*-infected mosquitoes remain to be assessed, such as whether the *Wolbachia* will still be capable of inhibiting the virus after repeated vertical transmission in the mosquitoes, whether the pathogens will gradually adapt to *Wolbachia*-infected host through mutations, or changes in mosquito itself can increase vectorial capacity despite of the presence of *Wolbachia* infection. Host, *Wolbachia*, and virus genetic evolution could all influence the long-term success of *Wolbachia* programs [22].

Elucidating the mechanisms of PI phenomenon will be of great importance in maximizing the effects of *Wolbachia*-based mosquito-control strategies, extending the sustainability of this method, quickly understanding and correctly solving problems that may arise in the future. Till now, the mechanism underlying PI is still not completely understood. Currently two major explanations have been proposed. One is that *Wolbachia* activates the mosquito innate immune responses, and thus-primed immune system helps the host to fight subsequent pathogen infections [14, 17, 23]. The other one is that *Wolbachia* competes with pathogens for nutrients such as lipids [24, 25].

Although existing studies suggest that the innate immune response may play a leading role in *Wolbachia* induced PI in mosquitoes, we should also notice that those studies were all based on artificially or naturally *Wolbachia*-infected host, using uninfected host as a control. Compared with the control group, the presence of *Wolbachia* in the infected host may both up-regulate host immune response and compete for nutrients. The effects of these two concomitant processes on the replication of pathogens are indistinguishable.

Alternatively, a comparison between infected populations may help to elucidate the role of immune responses in PI. To that end, we choose *Culex* mosquitoes in which *Wolbachia* is prevalent. *Culex* mosquitoes are an important vector of lymphatic filariasis and several viral pathogens, including West Nile virus [26]. The most prevalent *Culex* species in China is *Cx*. *pipiens pallens*. Our previous study [27] revealed that the bi-directional incompatibilities between naturally existent populations from different geographic locations were dependent on the presence of *Wolbachia*, i.e. they were *Wolbachia*-induced CI. For example, Nanjing (NJ) and Tangkou (TK) populations were naturally infected with bi-directionally incompatible *Wolbachia*. Based on the fact that *Wolbachia* is maternally inherited, in this study, we propose to cross preexisting host-*Wolbachia* symbioses obtained in Nanjing and Tangkou to create new host-*Wolbachia* symbioses. Comparing the transcriptomes in the old and new host-*Wolbachia* symbiotic combinations in which nutrient competition is constantly present, we aim to delineate the contribution of innate immune responses to PI in *Wolbachia*-infected mosquitoes.

## Materials and methods

### Mosquitoes

The *Cx*. *pipiens pallens* larvae were collected from Nanjing (NJ), Jiangsu Province(32°3'30.11"N, 118°47'47.28"E), and Tangkou (TK), Shandong Province(34°52'34.97"N, 117°22'53.69"E) from July to August in 2017. All collection was done on public land. After morphology identification, the larvae were then maintained in an insectary. Mosquitoes were kept at 28°C, 75% relative humidity and a photoperiod of 14h light: 10h darkness. Adult mosquitoes were fed 10% (w/v) glucose solution prior to blood meals [27].

### Tetracycline treatment

Tetracycline treatment to eliminate *Wolbachia* from *Culex* populations was carried out according to published methods [28]. Tetracycline (Amresco) at a concentration of 0.05 mg/ml was used for the treatment through both larval and pupal stages. Eggs were placed on tetracycline water solution to hatch. Surviving larvae were transferred to fresh tetracycline solution every 24 hours. A normal infusion was prepared in parallel and fed to larvae in tetracycline solution. After continuous tetracycline treatment for 6 generations, *Wolbachia*-negative *Culex* populations were established.

### Establishment of new host-Wolbachia symbioses

To separate virgin females and males, pupae from each population were put into 15 ml tubes with water for individual emergence. Then, male and female adults were raised in 30.5×30.5×30.5 cm cages. Females 1 day post-eclosion and males 2 days post-eclosion were used in crossing experiments. Each set of crossings included combination groups of *Wolbachia*-negative virgin males from TK with *Wolbachia*-positive virgin females from NJ(NJ♀×TK_tet_♂), *Wolbachia*-negative virgin males from NJ with *Wolbachia*-positive virgin females from TK(TK♀×NJ _tet_♂). While combinations of virgin males and females from the same populations as controls (NJ♀×NJ♂ and TK♀×TK♂). Females and males placed in the same cages were given 2 days to mate. Females were blood fed after mating, then the egg rafts were given 48 hours after oviposition to hatch. Females of the first filial generation of these crossings, namely NJ♀×TK_tet_♂, TK♀×NJ _tet_♂, NJ and TK were collected 2 days post-eclosion for RNA extraction and sequencing.

### RNA sequencing and analysis

Total RNA of 15 female mosquitoes of each group (NJ♀×NJ♂, NJ♀×TK_tet_♂, TK♀×TK♂ and TK♀×NJ_tet_♂) was extracted using TRIzol reagent (Thermo Fisher Scientific, USA) following the manufacturer’s protocol. cDNA library construction and sequencing were performed according to standard procedures by Beijing Genomics Institute (BGI-Shenzhen, China) using BGISEQ-500 platform. At least 60 Mb clean reads of sequencing were obtained for each sample. Since no genomic sequence in any database was available for *Cx*. *pipiens pallens*, Trinity [29] was used to perform de novo assembly with clean reads, then Tgicl [30] was used on cluster transcripts to remove abundance and retain Unigenes. After assembly, Unigenes functional annotation was performed with 7 functional databases (NR, NT, GO, KOG, KEGG, SwissProt and InterPro), then all the clean reads of each sample were mapped to the Unigenes with Bowtie2 [31] software and the gene expression levels were calculated with RSEM [32]. Based on the gene expression levels, the DEGs (differential expression genes) between samples or groups were identified with PossionDis [33] (Fold Change > = 2.00 and FDR< = 0.001). The DEGs were classified based on the GO annotation results and official classification. Pathway analysis was performed to provide further information on the DEGs’ biological functions. The DEGs were also subjected to KEGG pathway classification and functional enrichment. As a biological replicate of this experiment, total RNA of another 15 female mosquitoes from each group was extracted for cDNA library construction and sequencing. A total of eight libraries were sequenced and analyzed.

### Validation of immunity-related DEGs by real-time quantitative PCR

Each total RNA template was obtained from a pool of 5 female mosquitoes and extracted as described above. We generated three biological replicates for each group. For each biological replicate three independent total RNA templates were obtained. Totally, we have 3×3 total RNA templates for each group. The cDNA was synthesized with PrimeScript RT reagent kit (Takara, Otsu, Shiga, Japan) according to the manufacturer's protocol. PCR was performed on the LightCycler 96 Real-Time PCR System (Roche, Switzerland) using SYBR Green Master Mix kit (Roche, Switzerland). Primers specific for real-time quantitative PCR are listed in Table 1. We amplified 23 different genes from each template. Each gene amplication was carried out in triplicate. For each reaction, 10 μl of SYBR Green Master Mix was used, 1.0 μl of each primer solution at 10 μM and 8 μl of diluted cDNA were added. PCR cycling protocol was as follows: initial 50°C for 2 min, denaturation for 10 min at 95°C, followed by 40 cycles of 95°C for 15 s, 60°C for 1 min. The housekeeping gene Rps6 was used as an internal control and the data were analyzed with LightCycler 96 Software v1.1 (Roche, Switzerland). Quantitation of relative mRNA expression was calculated using 2^−ΔΔCt^ method [34]. Significance was determined based on comparison of the ΔCT of each gene in old and new host-*Wolbachia* symbioses using Student’s *t*-tests. *P<0.05; **P<0.01.Immunity-related DEGs were further analyzed with PathVisio software 3.3.0. obtained from wikipathways (WP3830_92694) which is based on the Toll and Imd Pathways in *Drosophila melanogaster*.

**Table 1:** Primer sequences used in real-time quantitative PCR.

Primer Name		Primer Sequence (5'→3')
RPS6	Sense	TGATTCGCTGTTGTATCGTGGA
	Antisense	GATGTTATTCGCACGCTTCG
WSP	Sense	TGCAAACAGTGTGGCAGCAT
	Antisense	ACCAACACCAACACCAACGTA
MODSP	Sense	AGAATTCCGCTTCTGCGACA
	Antisense	ACTCCGGATACACGATGGGA
GRASS	Sense	ACATCAATGGGTACACGCGG
	Antisense	GGAGTCGGTTCTCAAGGTCG
SPIRIT	Sense	GAGTCGATCGTGCTGCAAAA
	Antisense	GCAAACTCCCGCCACATTTC
SPZ1B	Sense	ATCGGCAAGGATTTTGACGC
	Antisense	GCGTTGCCATTTCCCTTCAG
TOLL	Sense	CCAATGAATGTGGTGGCGTT
	Antisense	TCCCAACATTCTGTGGCATCA
DIF	Sense	ACGGTCGAGATCAACAGTGC
	Antisense	GCTTGGCGTGACTGTAACCA
DEFA	Sense	TGGATTCGGCGTCAACGATA
	Antisense	CACACGCAAACCTTCTTGCC
EFFETE	Sense	GATTTGCTCACTTCCGGTCG
	Antisense	GACCTCCAGTATCCGCTTCC
IAP2	Sense	CTGGCCACCTTCGTCAACTG
	Antisense	GACCTCCCACTGGCCGATAA
TAK1	Sense	TCCCTTAACATTTCCAACGCC
	Antisense	CCAGGATGCTGTTGAGGGAT
JNK	Sense	GCGGATGTTTGGACTGTTCC
	Antisense	CCGATCATGGTCCAACTCCA
RELISH	Sense	CCGTACTACGACGACGGAAG
	Antisense	CGAAAGCGGAACTTGTCCAC
PSH	Sense	TTCATCCGGAGTACGACCCT
	Antisense	AAGCCCAACCACCTTGGAAA
SPE	Sense	CTGGACGTTGGAGTGGAAGA
	Antisense	CAGGCAAAGCGGAGAGATGA
GNBPB3	Sense	GTTGGCTGGCAATACGAACTG
	Antisense	AACGGCTCACCGAACTCCTC
GNBPB2	Sense	GACAGATGTACCGACGAGCC
	Antisense	ATTCAGAATTCGGGACGGGG
PGRP-LC	Sense	GTGGTCACAGCACGGAGTTTATT
	Antisense	TTCAGCTCATTTCCCTTGTCTATCT
IMD	Sense	GCTCGCTGAGCAAATTTACCAATTT
	Antisense	CGTTCCTTCCACAGCACCTTC
CECA	Sense	GCTGTTCGTCATCGTCCTG
	Antisense	CCCGTTTGCCAACTCCTT
CECB	Sense	ATTGTCATTCTGGCAGCCCT
	Antisense	CACTCGCTTGCCAGCTTTTT
NEC	Sense	ACCAAGCGTGAACTCTCCAA
	Antisense	AGTTGCCGTTCTCCTTCGTT

### Microbial challenge and survival experiments

Microbial challenge and survival experiments were performed in the same way as described in [35]. In brief, an acupuncture needle (0.20×25mm) was dipped into a concentrated overnight bacterial culture of Gram-negative (*Escherichia coli*) or Gram-positive (*Micrococcus luteus*) bacteria or sterile LB culture (negative control) and pricked mosquitoes (female 2 days post eclosion) in the rear part of the abdomen. For each mosquito population, three parallel groups with each group consisting of 15–20 adult females were inoculated per bacterial species [36]. A total of three biological replicates of the infection experiment were performed. Survival curves are significantly different between mosquitoes in old and new host-*Wolbachia* symbioses (compared using log-rank test).

### Statistical analysis

All statistical analyses were carried out using SPSS Statistics 17.0.

### Data accessibility

The data supporting the results of this article have been submitted to NCBI Sequence Read Archive (SRA) repository (Accession number: SRP155507). The materials and methods part has been submitted to protocols.io. (DOI:http://dx.doi.org/10.17504/protocols.io.xcafise)

## Results

### No significant change of Wolbachia density in hosts with hybrid genetic profiles

**Fig 1:**
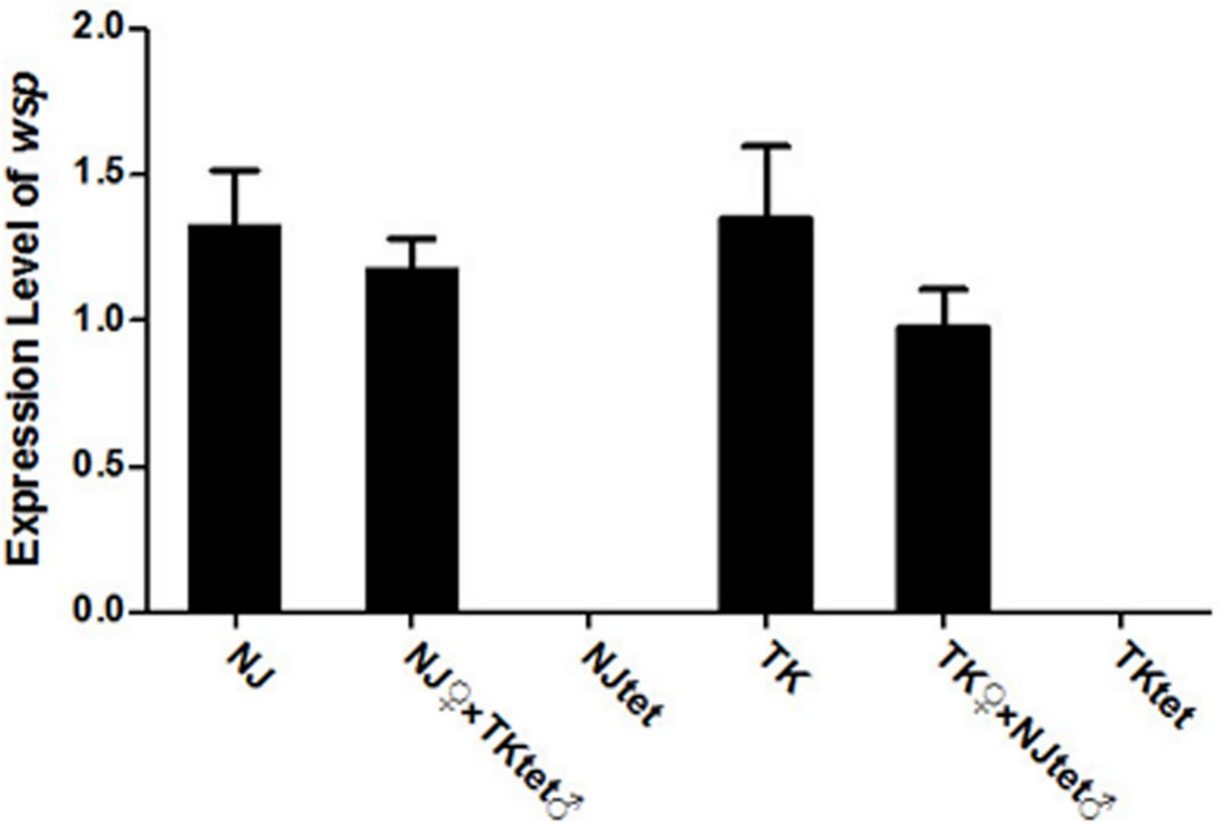
No significant change of *Wolbachia* density in hosts with hybrid genetic profiles. The expression of *wsp* gene was measured by real-time quantitative PCR in NJ♀×TK_tet_♂, TK♀×NJ_tet_♂, NJ_tet_, TK_tet_, NJ and TK virgin females at 2 days post-eclosion. *wsp* gene expression data were normalized with RPS6. The primer sequences are shown in Table 1. The bars indicate standard error. 2^−ΔΔCT^ method was used to calculate the expression level. Significance was determined based on comparison of the ΔCT using Student’s t-tests.

With continuous tetracycline treatment for 6 generations, we established *Wolbachia*-negative *Culex* populations TK_tet_ and NJ_tet_. To confirm the absence of *Wolbachia*, we conducted real-time quantitative PCR to quantify any residual *Wolbachia*-specific DNA. As shown in Fig 1, compared to that in TK and NJ populations, *wsp* (*Wolbachia* major surface protein) gene abundance decreased to an undetectable level in TK_tet_ (t = 5.424, df = 4, *P* = 0.0056)and NJ_tet_ populations(t = 6.749, df = 4, *P* = 0.0025). Regular PCR amplification of *wsp* using total DNA from TK_tet_ and NJ_tet_ as template also gave a negative result (data now shown). Then we conducted crossing experiments using *Wolbachia*-negative males and *Wolbachia*-positive females, and acquired NJ♀×TK_tet_♂ and TK♀×NJ_tet_♂ populations which represent novel host-*Wolbachia* symbioses (NJ *Wolbachia* in TK-NJ hybrid host or TK *Wolbachia* in TK-NJ hybrid host) compared to the original NJ and TK populations (NJ *Wolbachia* in NJ host or TK *Wolbachia* in TK host). Real-time quantitative PCR results showed that *Wolbachia* densities were not significantly changed in hosts with hybrid genetic profiles (for NJ♀ and NJ♀×TKtet♂ groups: t = 0.6536, df = 4, *P* = 0.5491, for TK♀ and TK♀×NJ_tet_♂ groups: t = 1.317, df = 4, *P* = 0.2581). (Fig 1)

### Comparative transcriptome analysis of the original and newly created host-Wolbachia symbioses

**Fig 2:**
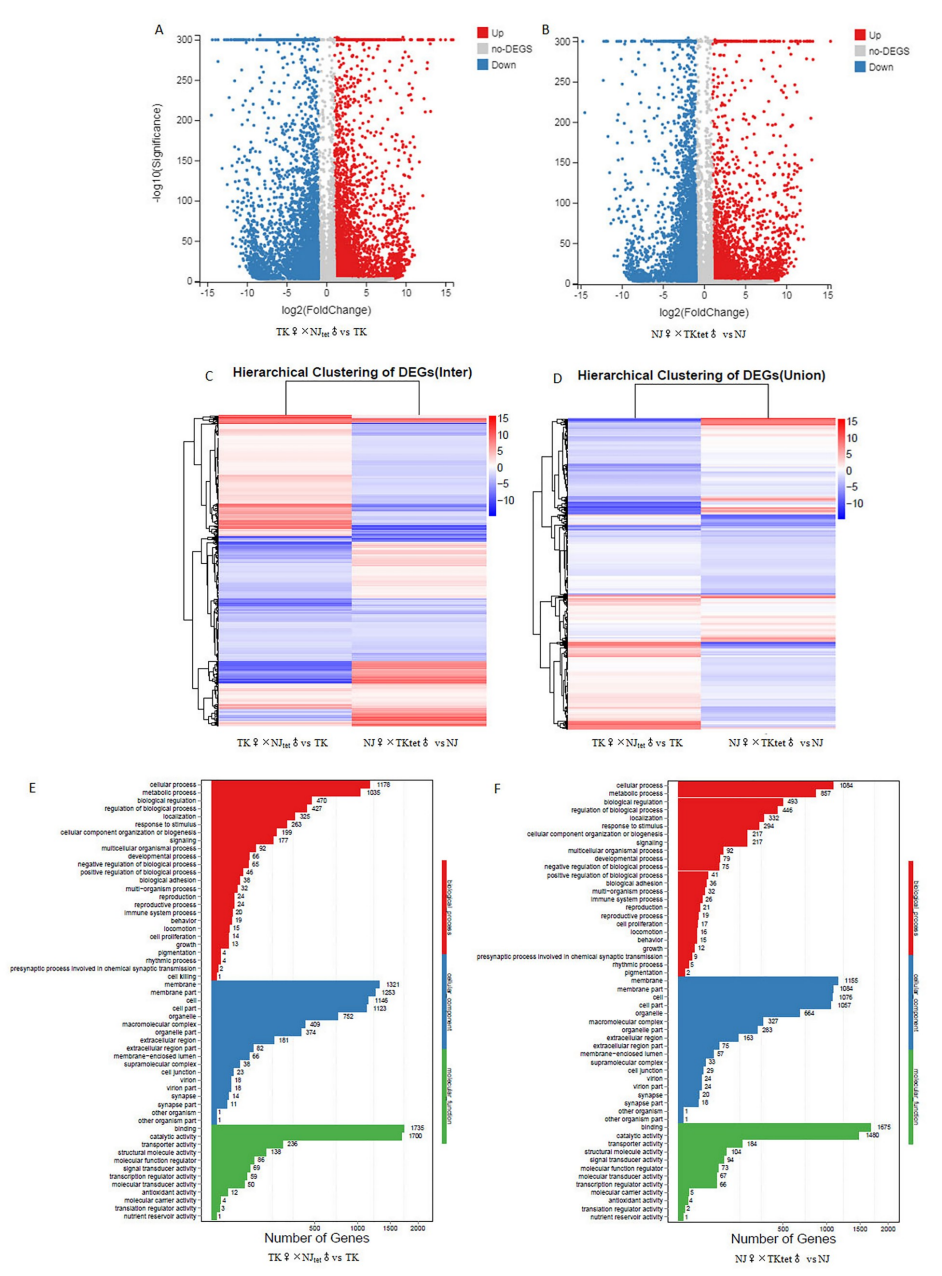
(A and B) Volcano plot of DEGs. The unigenes up- or down-regulated more than two-fold when compared between old and new host-*Wolbachia* symbioses are displayed in red or blue, respectively. Y axis represents -log10 transformed significance. X axis represents log2 transformed fold change. Red points represent up-regulated DEGs. Blue points represent down-regulated DEGs. Gray points represent non-DEGs. (C and D) Heatmap of hierarchical clustering of DEGs. X axis represents comparison for clustering analysis. Coloring indicates fold change (high: red, low: blue). (E and F) GO classification of DEGs. X axis represents number of DEG. Y axis represents GO term.

**Fig 3:**
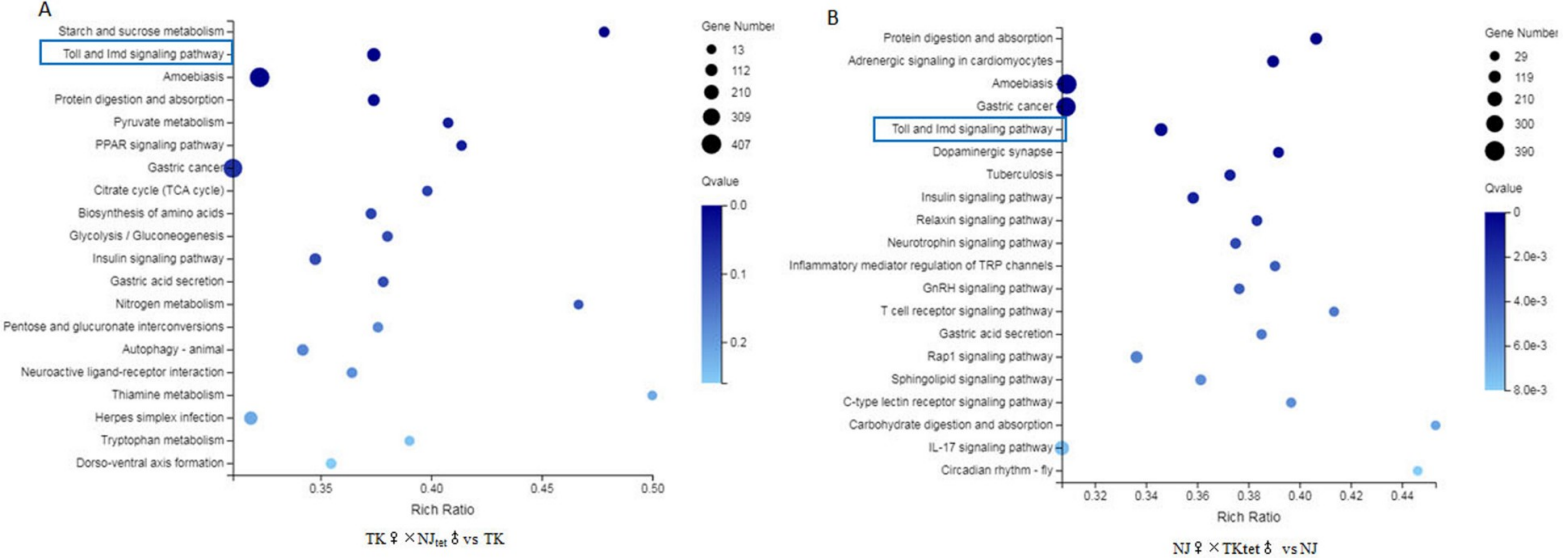
Up-regulation of Toll and IMD pathway genes in new host-*Wolbachia* symbioses. Pathway functional enrichment of DEGs. X axis represents enrichment factor. Y axis represents pathway name. The color indicates q value (high: white, low: blue), a lower q value indicates a more significant enrichment. Point size indicates DEG number (A bigger dot refers to a larger amount). Rich Factor refers to the value of enrichment factor, which is the quotient of foreground value (the number of DEGs) and background value (total Gene amount). A larger Rich Factor value indicates a higher level of enrichment.

cDNA libraries were sequenced from the original and newly created mosquito host-*Wolbachia* symbioses. 24,659 (NJ♀×TKtet♂), 26,777 (TK♀×NJ tet♂), 27,513 (NJ♀×NJ♂) and 28,123 (TK♀×TK♂) unigenes were generated. A total of 24,970 unigenes were annotated against the NCBI NR protein database, 15,598 in GO function categories, and 19,396 unigenes were mapped onto the canonical pathways in KEGG. The unigene expression in new host-*Wolbachia* symbioses was compared with original mosquito host based on the fragments per kilobase of transcript per million mapped reads (FPKM) value. TK♀×NJ_tet_♂ had 4,148 up-regulated unigenes and 5,036 down-regulated unigenes in comparison to the control TK♀×TK♂, and NJ♀×TK_tet_♂ had 2,712 up-regulated unigenes and 5,747 down-regulated unigenes in comparison to the control NJ♀×NJ♂ (Fig 2A and 2B). The intersection and union of the DEG heat map for the original and new host-*Wolbachia* symbioses are shown in Fig 2C and 2D. The identified DEGs were then assigned to the three standard subcategories of “molecular biological function”, “cellular component” and “biological process” in GO enrichment analysis (Fig 2E and 2F). In parallel, the unigenes were mapped onto the canonical pathways in KEGG to identify possible active biological pathways that contain DEGs. Twenty most significant DEGs in new vs. old host-*Wolbachia* symbioses are shown in Fig 3. RNA-seq data analysis of a biological replicate are presented in supplementary materials and shown in S1 and S2 Figs.

### Innate immune responses are elevated in hosts with hybrid genetic profiles

**Fig 4:**
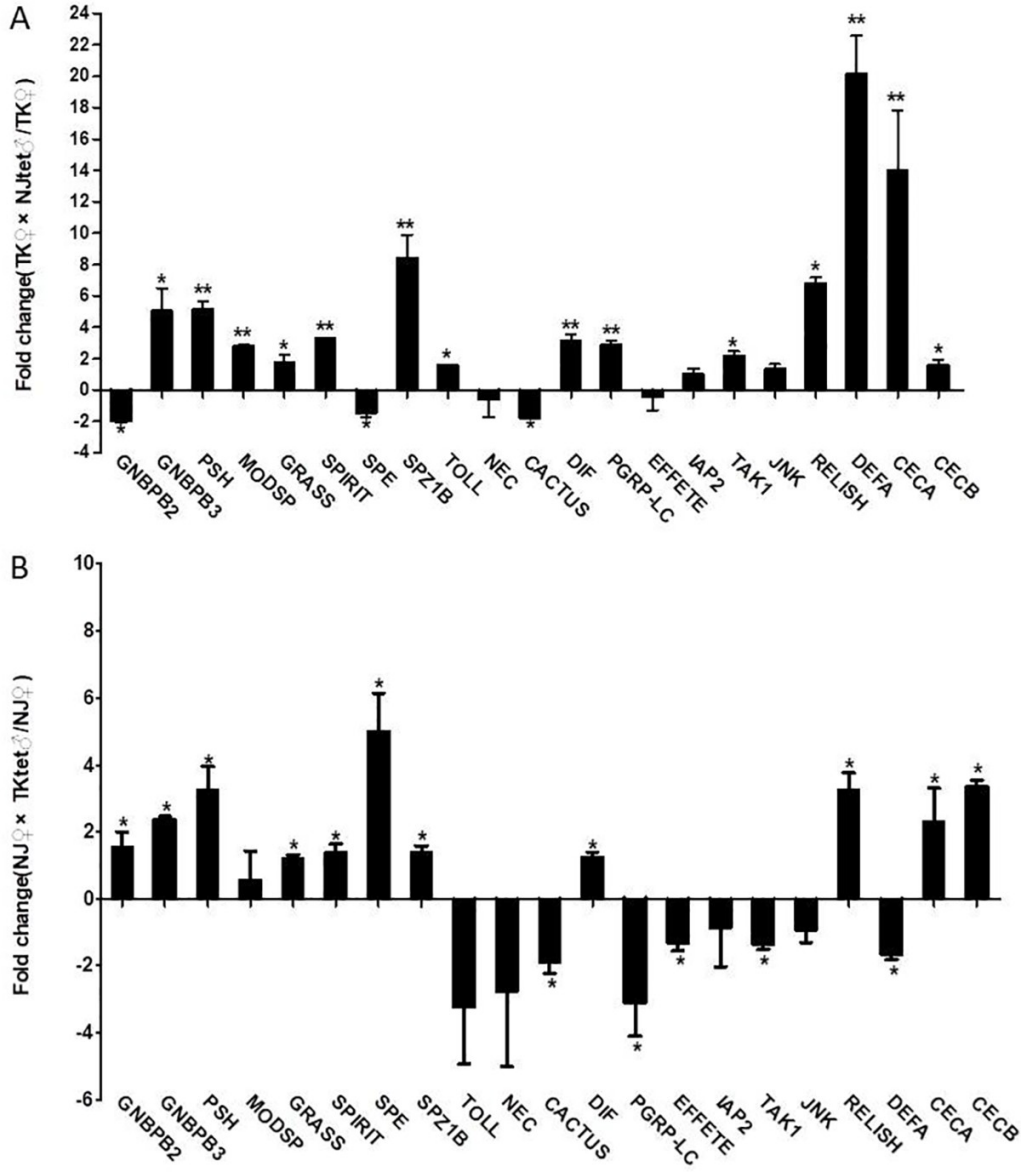
Survival curves of the mosquitoes post-challenge with *M. luteus* (a, c) or *E. coli* (b, d). For each mosquito population, three parallel groups of 15-20 adult females each were inoculated per bacterial species. A total of three biological replicates of the infection experiment were performed. Error bars indicate the standard error. Survival curves are significantly different between mosquitoes in old and new host-*Wolbachia* symbioses (compared using log-rank test).

**Fig 5:**
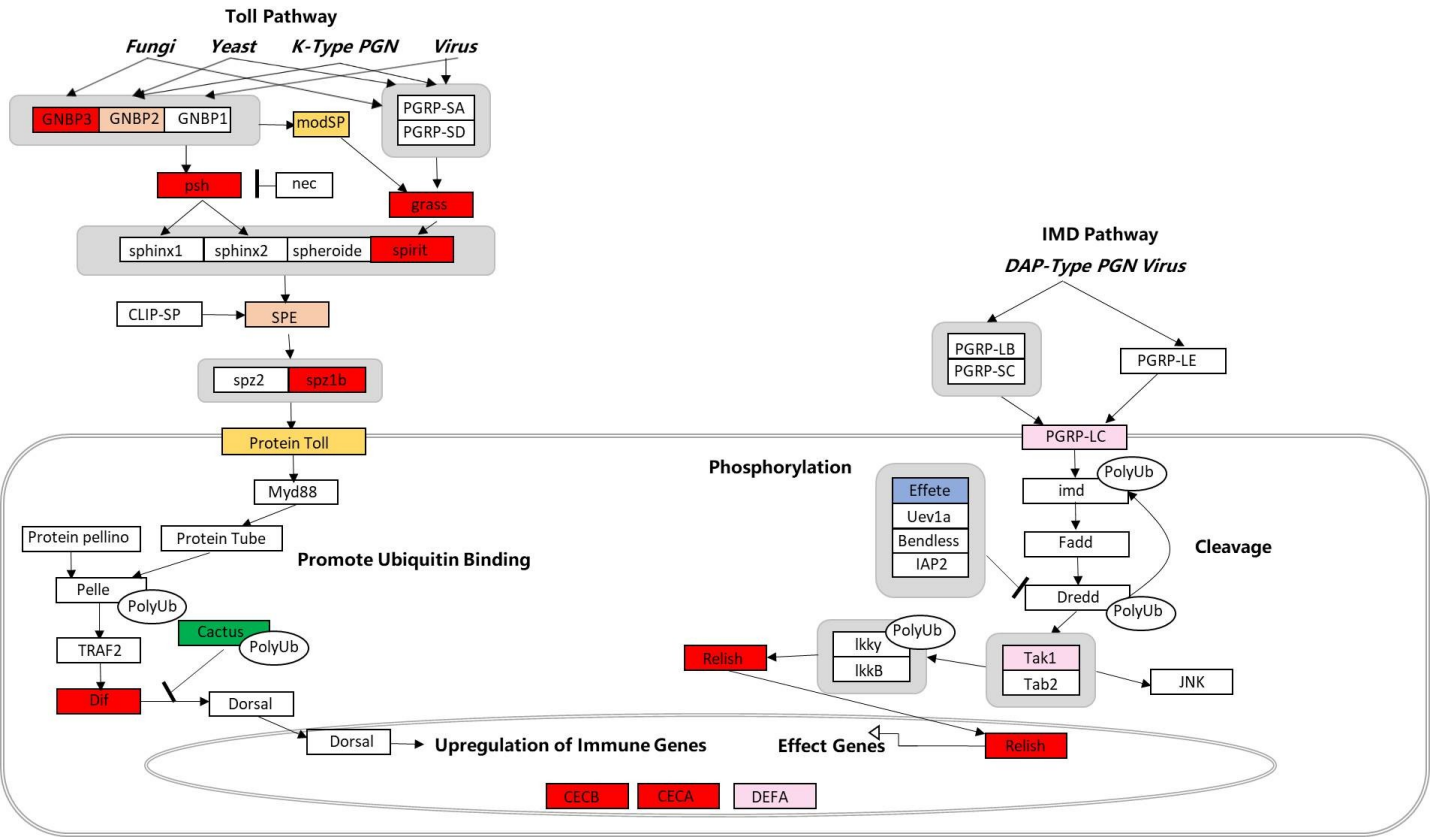
Regulation of putative Toll and Imd signaling pathway genes in hosts with hybrid genetic profiles. Red color indicates Toll and Imd signaling pathway genes up-regulated in both TK♀×NJ_tet_♂ (compare to TK♀) and NJ♀×TK_tet_♂ (compare to NJ♀) groups, pink color indicates up-regulated in TK♀×NJ_tet_♂ but down-regulated in NJ♀×TK_tet_♂ group, orange color indicates up-regulated in NJ♀×TK_tet_♂ but down-regulated in TK♀×NJ_tet_♂ group, green color indicates down-regulated in both TK♀×NJ_tet_♂ and NJ♀×TK_tet_♂ groups, yellow color indicates up-regulated only in TK♀×NJ_tet_ group, blue color indicates down-regulated only in NJ♀×TK_tet_♂ group, white indicates unfound. The pathway was built with PathVisio software 3.3.0 based on the Toll and IMD Pathways of *Drosophila melanogaster* downloaded from wikipathways (WP3830_92694).

Based on the transcriptome assays, we compared mosquito innate immune responses in the original and new host-*Wolbachia* symbioses. As shown in Fig 3 and S1 Table, Genes in Toll and the immune deficiency (Imd) signaling pathway were up-regulated in both TK♀×NJtet♂ (compare to TK) and NJ♀×TKtet♂ (compare to NJ) groups. The differential activations of immune responses in hosts of different genetic profiles to the same *Wolbachia* were confirmed by real-time PCR quantification of genes in the Toll and Imd pathways (Fig 4). Our results showed that Toll pathway genes, such as Gram-negative binding protein B3 (GNBPB3, for NJ♀ and NJ♀×TKtet♂ groups: t = 2.136, df = 4, *P* = 0.0497, for TK♀ and TK♀×NJ_tet_♂ groups: t = 3.214, df = 4, *P* = 0.0162), serine protease persephone (PSH, for NJ♀ and NJ♀×TKtet♂ groups: t = 3.192, df = 4, *P* = 0.0166, for TK♀ and TK♀×NJ_tet_♂ groups: t = 8.187, df = 4, *P* = 0.0019), gram-positive specific serine protease (GRASS, for NJ♀ and NJ♀×TKtet♂ groups: t = 2.924, df = 4, *P* = 0.0215, for TK♀ and TK♀×NJ_tet_♂ groups: t = 2.560, df = 4, *P* = 0.0416), serine protease immune response integrator (SPIRIT, for NJ♀ and NJ♀×TKtet♂ groups: t = 2.224, df = 4, *P* = 0.0451, for TK♀ and TK♀×NJ_tet_♂ groups: t = 5.411, df = 4, *P* = 0.0028), Spaetzle-like cytokine 1B (SPZ1B, for NJ♀ and NJ♀×TKtet♂ groups: t = 2.628, df = 4, *P* = 0.0292, for TK♀ and TK♀×NJ_tet_♂ groups: t = 8.305, df = 4, *P* = 0.0018), and Dorsal-related immunity factor (DIF, for NJ♀ and NJ♀×TKtet♂ groups: t = 2.933, df = 4, *P* = 0.0213, for TK♀ and TK♀×NJ_tet_♂ groups: t = 6.436, df = 4, *P* = 0.0038) were up-regulated in the new host-*Wolbachia* symbioses. Developmental protein cactus (CACTUS) was down-regulated in the new host-*Wolbachia* symbioses(for NJ♀ and NJ♀×TKtet♂ groups: t = 2.550, df = 4, *P* = 0.0316, for TK♀ and TK♀×NJ_tet_♂ groups: t = 2.685, df = 4, *P* = 0.00275). We also observed that genes representing the Imd pathway, such as nuclear factor NF-κB p105 subunit (RELISH) were up-regulated in the new host-*Wolbachia* symbioses(for NJ♀ and NJ♀×TKtet♂ groups: t = 2.649, df = 4, *P* = 0.0285, for TK♀ and TK♀×NJ_tet_♂ groups: t = 3.562, df = 4, *P* = 0.0189). In addition to the majority genes of Toll and Imd pathways that were up-regulated in new host-*Wolbachia* symbioses, some genes did not show consistent upregulation. For example, Gram-negative binding protein B2 (GNBPB2) and Spaetzle-processing enzyme (SPE) were up-regulated in NJ♀×TKtet♂ while down-regulated in TK♀×NJtet♂(GNBPB2:for NJ♀ and NJ♀×TKtet♂ groups: t = 2.893, df = 4, *P* = 0.0222, for TK♀ and TK♀×NJ_tet_♂ groups: t = 3.209, df = 4, *P* = 0.0163; SPE: for NJ♀ and NJ♀×TKtet♂ groups: t = 2.289, df = 4, *P* = 0.0420, for TK♀ and TK♀×NJ_tet_♂ groups: t = 3.443, df = 4, *P* = 0.0131). Peptidoglycan recognition protein-lc (PGRP-LC) and TGF-Beta-Activated Kinase-1 (TAK1) were up-regulated in TK♀×NJtet♂ while down-regulated in NJ♀×TKtet♂(PGRP-LC:for NJ♀ and NJ♀×TKtet♂ groups: t = 3.141, df = 4, *P* = 0.0174, for TK♀ and TK♀×NJ_tet_♂ groups: t = 7.730, df = 4, *P* = 0.0023; TAK1: for NJ♀ and NJ♀×TKtet♂ groups: t = 2.888, df = 4, *P* = 0.0223, for TK♀ and TK♀×NJ_tet_♂ groups: t = 2.569, df = 4, *P* = 0.0403). Proteins Toll and modular serine protease (MODSP) were up-regulated only in TK♀×NJtet♂(t = 9.633, df = 4, *P* = 0.0012). Effete (EFFETE) was down-regulated only in NJ♀×TKtet♂(t = 3.254, df = 4, *P* = 0.0156). Transcripts of the antimicrobial peptide genes (effector genes) cecropin A (CECA) and cecropin B (CECB) were up-regulated in the new host-*Wolbachia* symbioses(CECA:for NJ♀ and NJ♀×TKtet♂ groups: t = 3.561, df = 4, *P* = 0.0118, for TK♀ and TK♀×NJ_tet_♂ groups: t = 8.849, df = 4, *P* = 0.0015; CECB: for NJ♀ and NJ♀×TKtet♂ groups: t = 3.490, df = 4, *P* = 0.0251, for TK♀ and TK♀×NJ_tet_♂ groups: t = 3.785, df = 4, *P* = 0.0323). While defensin A (DEFA) was up-regulated in TK♀×NJtet♂(t = 7.137, df = 4, *P* = 0.0028), it was down-regulated in NJ♀×TKtet♂(t = 3.323, df = 4, *P* = 0.0146). An analysis of the expression changes of Toll and Imd signaling pathway-related unigenes within new and old host-*Wolbachia* symbioses is given in Fig 5.

### Microbial challenge and survival experiments

**Fig 6:**
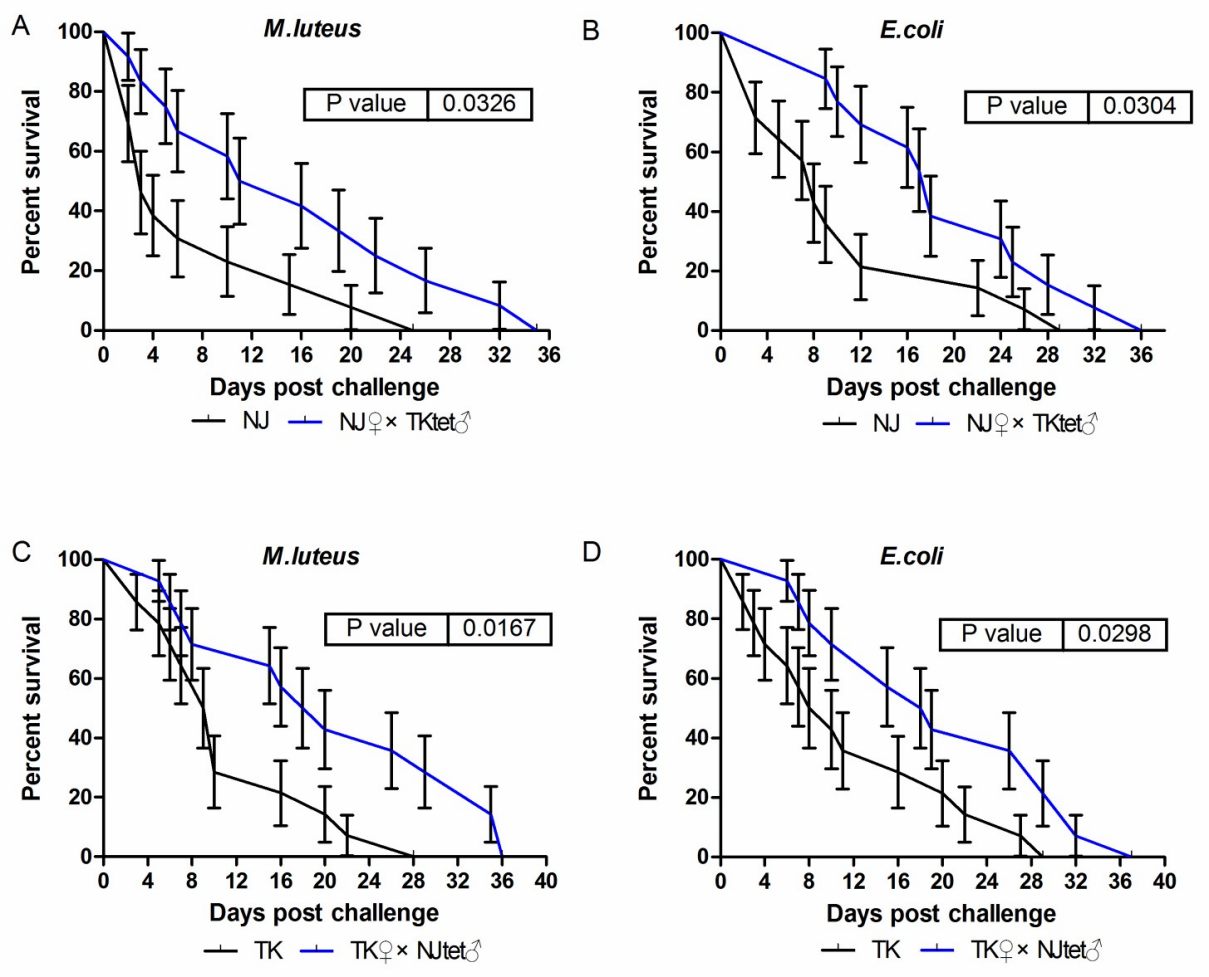
Survival curves of the mosquitoes post-challenge with *E*. *coli* (a) or *M*. *luteus* (b). For each mosquito population, three parallel groups of 15–20 adult females each were inoculated per bacterial species. A total of three biological replicates of the infection experiment were performed. Error bars indicate the standard error. Survival curves are significantly different between mosquitoes in old and new host-*Wolbachia* symbioses (compared using log-rank test).

Toll and Imd pathways are expected to protect mosquitoes from Gram-positive and Gram-negative bacterial infections respectively. Our results showed that both Toll and Imd pathways were up-regulated in new host-*Wolbachia* symbioses. To test if the up-regulation of these pathways can help mosquitoes to fight pathogen infections, we challenged mosquitoes in old and new host-*Wolbachia* symbioses with Gram-negative bacteria (*Escherichia coli*) and Gram-positive bacteria (*Micrococcus luteus*). Results showed that mosquitoes in new host-*Wolbachia* symbioses had higher survival rate than in old host-*Wolbachia* symbioses when challenged with either *E*. *coli* (P<0.05, for NJ♀ and NJ♀×TKtet♂ groups: chi square = 4.685, df = 1, *P* = 0.0304, for TK♀ and TK♀×NJ_tet_♂ groups: chi square = 4.395, df = 1, *P* = 0.0298) or *M*. *luteus* (P<0.05, for NJ♀ and NJ♀×TKtet♂ groups: chi square = 4.565, df = 1, *P* = 0.0326, for TK♀ and TK♀×NJ_tet_♂ groups: chi square = 5.730, df = 1, *P* = 0.0167) (Fig 6).

## Discussion

Population replacement aimed at *Wolbachia*-mediated PI is moving from benchtop to the field. Elucidation of PI mechanism may help to augment the efficacy of *Wolbachia*-based vector control, prolong its usage, and expedite comprehension of and solutions to unexpected problems in future practice. Insects have established a highly efficient innate immune system to distinguish between self and non-self molecules and resist infections. Host innate immune system recognizes pathogen-associated molecular patterns (PAMPs) via pattern-recognition receptors (PRRs) and initiates a cascade of responses [37]. PRR signaling is thought to be critical for the host to fight pathogens [38]. Studies in *Drosophila melanogaster* have shown that two main PRRs, Toll and Imd, are involved in arthropod immune responses. Gram-positive bacteria trigger the Toll pathway, fungi and Gram-negative bacteria trigger the Imd pathway, mediating innate immune responses and resulting in the production of antimicrobial peptides (AMPs) [39].

In mosquitoes, PI has been most thoroughly characterized in *Aedes aegypti*. Xi *et al*. propose that *Wolbachia* infection activates the innate immune response of *Ae*. *aegypti* by up-regulating the level of Toll pathway genes and the expression of antimicrobial peptides such as defensins, which enables mosquitoes to resist DENV. They found that when the Toll pathway inhibitor cactus gene was silenced, the extent of dengue infection in mosquitoes was reduced by 4.0-fold. When the Toll pathway was inactivated by silencing myd88, the virus load in mosquitoes increased 2.7 times compared to the control group [14, 23]. They also found that the elevation of reactive oxygen species (ROS) was a result of *Wolbachia* infection and was involved in the activation of the Toll pathway. Toll activation leads to the expression of antioxidants to alleviate oxidative stress and, as a “side-effect”, increases antimicrobial peptide production resulting in an enhanced resistance to pathogen infections [40]. Kambris *et al*. observed up-regulated immune genes in *Anopheles gambiae* somatically infected with *Wolbachia* and highly significant reductions in *Plasmodium* infection intensity. This effect was diminished after knockdown of TEP1 gene [17]. A different explanation of PI is that both *Wolbachia* and viruses such as DENV are heavily dependent on host lipids and other resources for survival, and a potential competitive effect could contribute to PI [24, 25]. Schultz *et al*. found that infection with *Wolbachia* inhibited the replication of ZIKV in mosquito cell lines, and increased supply of cholesterol moderately restored the replication of ZIKV [41]. However, there lacks reported research that extends this finding to adult mosquitoes.

While these previous studies on PI mechanism provided insightful information, they fell short of pinpointing the causes. In these studies, at least two coexistent factors were confounding each other, i.e., induction of innate immunity and competition for nutrients could both be effected by the presence of *Wolbachia* that was artificially introduced. It was difficult to rule out one of the two plausible explanations. In this study, we used preexisting host-*Wolbachia* symbioses (NJ *Wolbachia*—NJ mosquito & TK *Wolbachia*—TK mosquito) obtained in Nanjing and Tangkou to create mosquito populations representing new host-*Wolbachia* symbioses NJ♀×TK_tet_♂ and TK♀×NJ_tet_♂. In the new and original mosquito populations, *Wolbachia* was always existent, so that nutrient competition was constantly present. Our results showed that *Wolbachia* densities in the new mosquito populations did not change significantly. Thus, comparing the new and old host-*Wolbachia* symbioses, we can exclude nutrient competition factor and focus on the contribution of innate immunity. To find out if host immune system was activated by *Wolbachia* in altered host genetic background, we compared the transcriptomes in the old and new mosquito populations. Our results showed that both genes in Toll and those in Imd signaling pathways were up-regulated in new host-*Wolbachia* symbioses, indicating that *Wolbachia* may induce stronger immune responses in a new host than in the original host.

As initially reported in *D*. *melanogaster*, Toll does not directly recognize PAMPs in insects. Instead, PAMPs are detected by PGRPs (peptidoglycan-recognition proteins) and GNBPs (Gram negative-binding proteins) which activate proteolytic enzymes, leading to the cleavage thus activation of cytokine Spaetzle. Spaetzle binding crosslinks the ectodomains of Toll, and activates Toll receptor. Through the adaptor proteins MYD88, Tube and Pelle, Toll can then activate NF-kB protein DIF in immune-responsive tissues by dissociating DIF from the ankyrin-repeat inhibitory protein Cactus, leading to the production of AMPs [42]. Our transcriptome results showed that cecropin B was up-regulated in both TK♀×NJ_tet_♂ and NJ♀×TKtet♂, while cecropin A and defensin A were up-regulated in TK♀×NJ_tet_♂ but down-regulated in NJ♀×TKtet♂ (S1 Table). When further tested with real-time RT-PCR, only defensin A was consistently up-regulated in TK♀×NJ_tet_♂ and down-regulated in NJ♀×TKtet♂, both cecropin A and B were up-regulated in new host-*Wolbachia* symbioses (Fig 4). Although post-translational regulations (e.g. nuclear translocation) of upstream factors may be sufficient to induce the transcription of AMPs, both transcriptome and real-time RT-PCR results showed that GNBPB3, PSH, GRASS, SPIRIT, SPZ1B and DIF were all up-regulated in the new host-*Wolbachia* symbioses. Toll pathway inhibitor CACTUS was down-regulated in the new host-*Wolbachia* symbioses.

Imd pathway can be triggered by ligand binding to PGRP-LC [43]. The activation signal is transduced through intracellular adaptor IMD protein into two downstream branches. One branch has TAK1 acting as the downstream factor of Imd/FADD, which in turn activates IKK-β and IKK-γ homologues and directs phosphorylation of NF-kB transcription factor Relish. Activated Relish then translocates to the nucleus and promotes the transcription of AMPs [44]. The other branch activates the transcription factor AP-1 via JNK signaling [45, 46]. As some factors in Toll pathway, RELISH in Imd pathway was up-regulated in the new host-*Wolbachia* symbioses.

As a result of Toll and Imd pathway activation, cecropin A and B were consistently up-regulated as the host was replaced with a different genetic background. Cecropin A and B up-regulation are correlated with improved protection against challenge infections of bacteria. In contrast, defensin A was only up-regulated in TK♀×NJtet♂ and not in NJ♀×TKtet♂. One possible explanation is that an interplay between Toll and Imd pathways with participation of other factors results in the change in defensin A expression. Different *Wolbachia* may activate these pathways differentially and the balance between them determines if defensin A is up-regulated or down-regulated. It is also possible that different strains of *Wolbachia* have different sensitivities to defensin A, and those strains such as the one from Nanjing may have evolved more effective means to selectively down-regulate defensin A to assure their survival. This would be consistent with previous findings that not all strains of *Wolbachia* are equally susceptible to host immune responses [47]. Our results also showed, unlike cecropin A, defensin A was not correlated with protection against bacterial infections. These results are consistent with previous studies. For example, in a report by Pan *et al*., *Ae*. *Aegypti* infected with *w*AlbB showed defensin A up-regulation in the midgut but down-regulation in the rest of carcass [48].

In this study, the up-regulation of both Toll and Imd signaling pathways was not sufficient to significantly reduce the density of *Wolbachia* observed in the new hosts. It is unknown if this reflects a lack of enough genetic differences between the mosquitoes and between the *Wolbachia* strains. It is also unknown if an elevated overall immune response is able to suppress *Wolbachia* activity without altering its density. Nevertheless, the presence of *Wolbachia* helps to maintain the nonsterilizing immunity. Because the downstream effectors are not target-specific, the activated immune responses can also affect some pathogens. This has been tested in our challenge bacterial infections. When artificially infected with Gram-positive and Gram-negative bacteria, mosquitoes in new host-*Wolbachia* symbioses have significantly higher survival rates than the mosquitoes in original host-*Wolbachia* symbioses. Whether a similar effect can be observed in viral infections remains to be answered. There have been a number of reports on the contribution of innate immunity to the blocking of viral replications in insects [23, 40, 49]. Xi *et al*. reported that Toll pathway in *Aedes aegypti* controls dengue infection [23]. In a *Drosophila* model, Rancès *et al*. demonstrated that Toll pathway has an inhibitory effect on dengue in the presence or absence of *Wolbachia*, although neither Toll nor Imd pathway is necessary for *Wolbachia*-induced inhibition [47]. Because a host deficient in both Toll and Imd has not been tested, and other pathways such as JAK-STAT have been reported to suppress dengue replication, it remains possible that at least one of the Toll and Imd pathways has to be in place in order for *Wolbachia* to inhibit the viral replication [50]. In our study, both Toll and Imd were up-regulated by *Wolbachia* in new host genetic backgrounds. Whether these up-regulations will result in enhanced resistance to viral infections warrants future investigation.

In our study, *Wolbachia* was constantly present, so a competition for nutrients was also constitutive. In addition, *Wolbachia* densities in the original and new hosts were comparable, so the levels of nutrient deprivation would be comparable. It was unlikely that nutrient competition caused the difference in inhibition of pathogen proliferation and improvement of host survival. Instead, the elevated immune responses, likely induced by a “mismatch” between host and *Wolbachia* hence stronger antigen recognition, were responsible for the protection against subsequent infections. An immunity-mediated PI can also better explain the fact that naturally *Wolbachia*-infected insects retain their vectorial capacity. For example, *Aedes albopictus* is naturally infected with *Wolbachia*, but it can still transmit a variety of pathogens including dengue. In these hosts, native *Wolbachia* may have been recognized as self as a result of co-evolution. After all, immune responses induced by *Wolbachia* cause stress in the host and may deem undesirable in the absence of more pathogenic infections. An alternative explanation for natural *Wolbachia* infection not inducing PI is a reduced density and a more restricted tropism in the native hosts such as *Aedes fluviatilis*[7].

While the observed difference in resistance to bacteria is most likely caused by immunity, a contribution from nutritional factors to PI cannot be ruled out. It is possible that nutrient competition results in certain level of inhibition in all the mosquito hosts, and immune responses provide a further enhancement in those new hosts. In *Drosophila melanogaster*, *Wolbachia* has been found to cause virus interference without inducing overt up-regulation of immunity [51, 52]. At least for viral infections, *Wolbachia* can assert inhibition by depriving the host cells of essential nutrients.

By comparing mosquitoes that are all infected with *Wolbachia*, our study demonstrates the contribution of host innate immunity to PI phenomenon. Similar studies may be carried out using other genera of mosquitoes that are medically more important, such as *Anopheles* and *Aedes*. The elucidation of an immunity nature of PI is important to guide future practice. For example, *Wolbachia* may be genetically engineered to be more immunogenic. In current vector population replacement measures, it is difficult to predict how long the released insects will remain refractory to pathogen infections. In the event that these insects do acquire increased vectorial capacity, a possible solution may be to re-introduce a new strain of *Wolbachia*. Perhaps it is desired to search and isolate more strains of *Wolbachia*, and test more host-*Wolbachia* symbioses for future applications. Our results also suggest *Wolbachia*-based PI may be applied to naturally *Wolbachia*-infected mosquito populations, and extend to the control of a broader range of mosquito-borne diseases. A competition for nutrient may still be effected by *Wolbachia*, but this does not negate the potential of immunity-based strategies. Future practice may even forego the use of *Wolbachia* and focus on the introduction of non-self antigens into the genome of vector insects using transgenic techniques. Potential advantages of transgenic modification of host genome may include less technical difficulty and increased stability. For some insects, a stable *Wolbachia* infection may be difficult to achieve, such as in *Anopheles gambiae*. An immunogen-expressing transgene in vector genome may also be more stable since it is not subject to elimination due to chemical exposure.

### Accession numbers

The GenBank (http://www.ncbi.nlm.nih.gov/Genbank) accession numbers for sequences mentioned in the paper are: RPS6(XM_001848257.1), WSP(JX050186.1), GNBPB2(XM_001845757.1), GNBPB3(XM_001845228.1), PSH (XM_001868422.1), MODSP(XM_001849027.1), GRASS(XM_001844187.1), SPIRIT(XM_001842673.1), SPE (XM_001848834.1), SPZ1B (XM_001848360.1), TOLL (XM_001847119.1), NEC (XM_001866644.1), CACTUS (XM_001846332.1), DIF (XM_001844026.1), PGRP-LC (XM_001848006.1), EFFETE (XM_001845858.1), IAP2 (XM_001869624.1), TAK1 (XM_001848067.1), JNK (XM_001842775.1), RELISH (XM_001862241.1), DEFA (XM_001842893.1), CECA (XM_001861705.1), CECB (XM_001846866.1)

## Supporting information

S1 Fig Volcano plot, heatmap of hierarchical clustering and GO classification of DEGs. As a biological replicate, total RNA of another 15 female mosquitoes of each group was extracted. cDNA library construction and sequencing were performed as the first time. At least 60 Mb clean reads of sequencing were obtained for each sample.35,236 (NJ♀×TKtet♂), 34,965 (TK♀×NJ tet♂), 34,845 (NJ♀×NJ♂) and 34,708 (TK♀×TK♂) unigenes were generated. A total of 28,476 unigenes were annotated against the NCBI NR protein database, 16,973 in GO function categories, and 21,332 unigenes were mapped onto the canonical pathways in KEGG. (A and B) Volcano plot of DEGs. The unigenes up- or down-regulated more than two-fold when compared between old and new host-*Wolbachia* symbioses are displayed in red or blue, respectively. Y axis represents -log10 transformed significance. X axis represents log2 transformed fold change. Red points represent up-regulated DEGs. Blue points represent down-regulated DEGs. Gray points represent non-DEGs. TK♀×NJtet♂ had 5,742 up-regulated unigenes and 4,143 down-regulated unigenes in comparison to the control TK♀×TK♂, and NJ♀×TKtet♂ had 4,226 up-regulated unigenes and 4,122 down-regulated unigenes in comparison to the control NJ♀×NJ♂. (C and D) The intersection and union of the DEG heat map for the original and new host-*Wolbachia* symbiosis. X axis represents comparison for clustering analysis. Coloring indicates fold change (high: red, low: blue). (E and F) The identified DEGs were then assigned to the three standard subcategories of “molecular biological function”, “cellular component” and “biological process” in GO enrichment analysis. X axis represents number of DEG. Y axis represents GO term.
(TIF)

S2 Fig Up-regulation of Toll and IMD pathway genes in new host-*Wolbachia* symbioses. In parallel, the unigenes were mapped onto the canonical pathways in KEGG to identify possible active biological pathways of DEGs in biological replicate of RNA sequencing experiment. Twenty most significant DEGs in new vs. old host-*Wolbachia* symbiosis are shown here. X axis represents enrichment factor. Y axis represents pathway name. The color indicates q value (high: white, low: blue), a lower q value indicates a more significant enrichment. Point size indicates DEG number (A bigger dot refers to a larger amount). Rich Factor refers to the value of enrichment factor, which is the quotient of foreground value (the number of DEGs) and background value (total Gene amount). A larger Rich Factor value indicates a higher level of enrichment.
(TIF)

S1 Table Up-regulated Toll and Imd signaling pathway genes in new host-*Wolbachia* symbioses. (XLSX)

## Abbreviations

PI: pathogen interference
CI: cytoplasmic incompatibility
PCR: polymerase chain reaction
