# Supplementary figures and images for: Wolbachia limits pathogen infections through induction of host innate immune responses
Source: PLoS One. 2020 Feb 20;15(2):e0226736. doi: 10.1371/journal.pone.0226736 (PMC7032688; doi:10.1371/journal.pone.0226736)

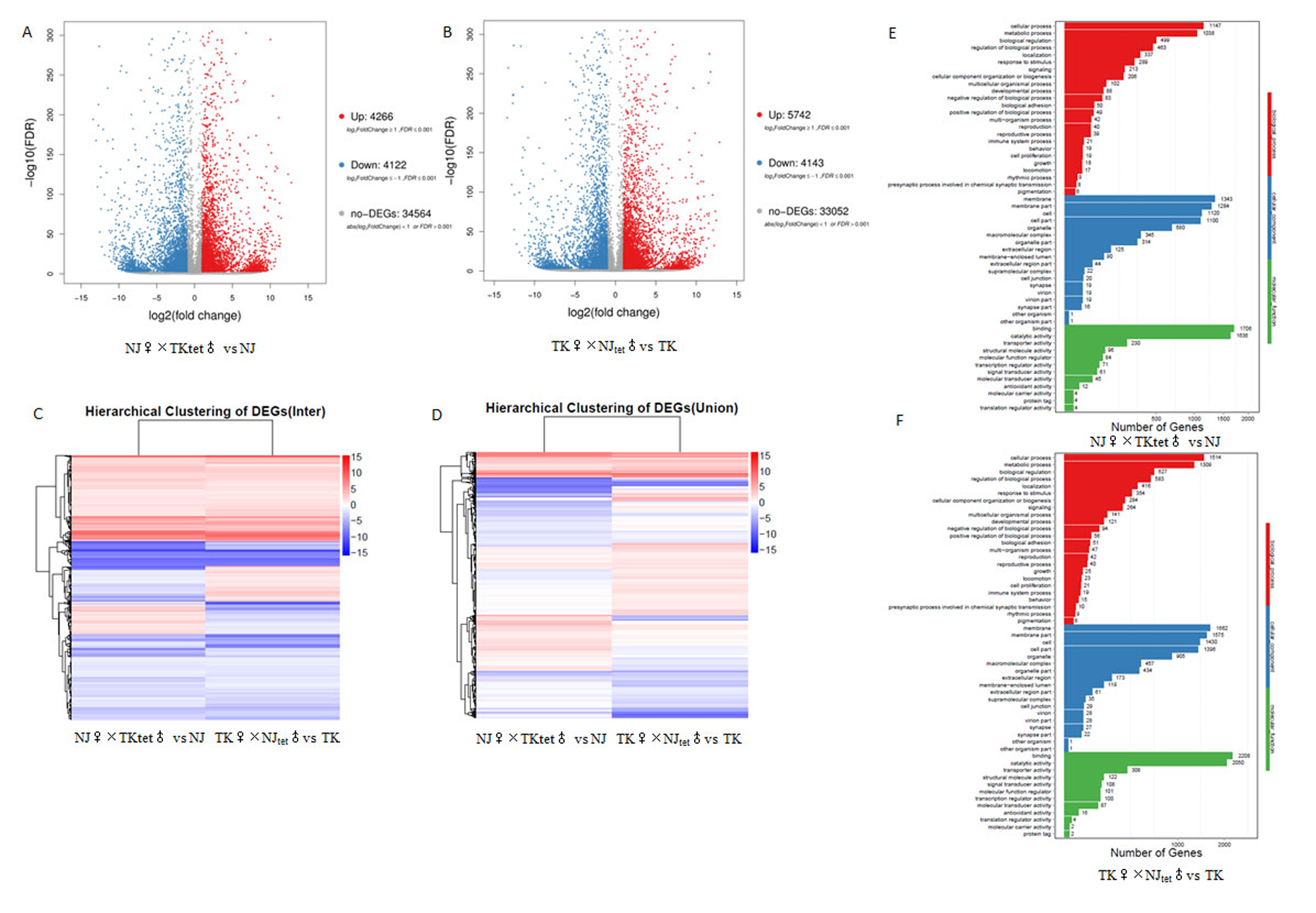

Supplement: S1 Fig — As a biological replicate, total RNA of another 15 female mosquitoes of each group was extracted. cDNA library construction and sequencing were performed as the first time. At least 60 Mb clean reads of sequencing were obtained for each sample.35,236 (NJ♀×TKtet♂), 34,965 (TK♀×NJ tet♂), 34,845 (NJ♀×NJ♂) and 34,708 (TK♀×TK♂) unigenes were generated. A total of 28,476 unigenes were annotated against the NCBI NR protein database, 16,973 in GO function categories, and 21,332 unigenes were mapped onto the canonical pathways in KEGG. (A and B) Volcano plot of DEGs. The unigenes up- or down-regulated more than two-fold when compared between old and new host-Wolbachia symbioses are displayed in red or blue, respectively. Y axis represents -log10 transformed significance. X axis represents log2 transformed fold change. Red points represent up-regulated DEGs. Blue points represent down-regulated DEGs. Gray points represent non-DEGs. TK♀×NJtet♂ had 5,742 up-regulated unigenes and 4,143 down-regulated unigenes in comparison to the control TK♀×TK♂, and NJ♀×TKtet♂ had 4,226 up-regulated unigenes and 4,122 down-regulated unigenes in comparison to the control NJ♀×NJ♂. (C and D) The intersection and union of the DEG heat map for the original and new host-Wolbachia symbiosis. X axis represents comparison for clustering analysis. Coloring indicates fold change (high: red, low: blue). (E and F) The identified DEGs were then assigned to the three standard subcategories of “molecular biological function”, “cellular component” and “biological process” in GO enrichment analysis. X axis represents number of DEG. Y axis represents GO term. (TIF) [file pone.0226736.s001.tif]

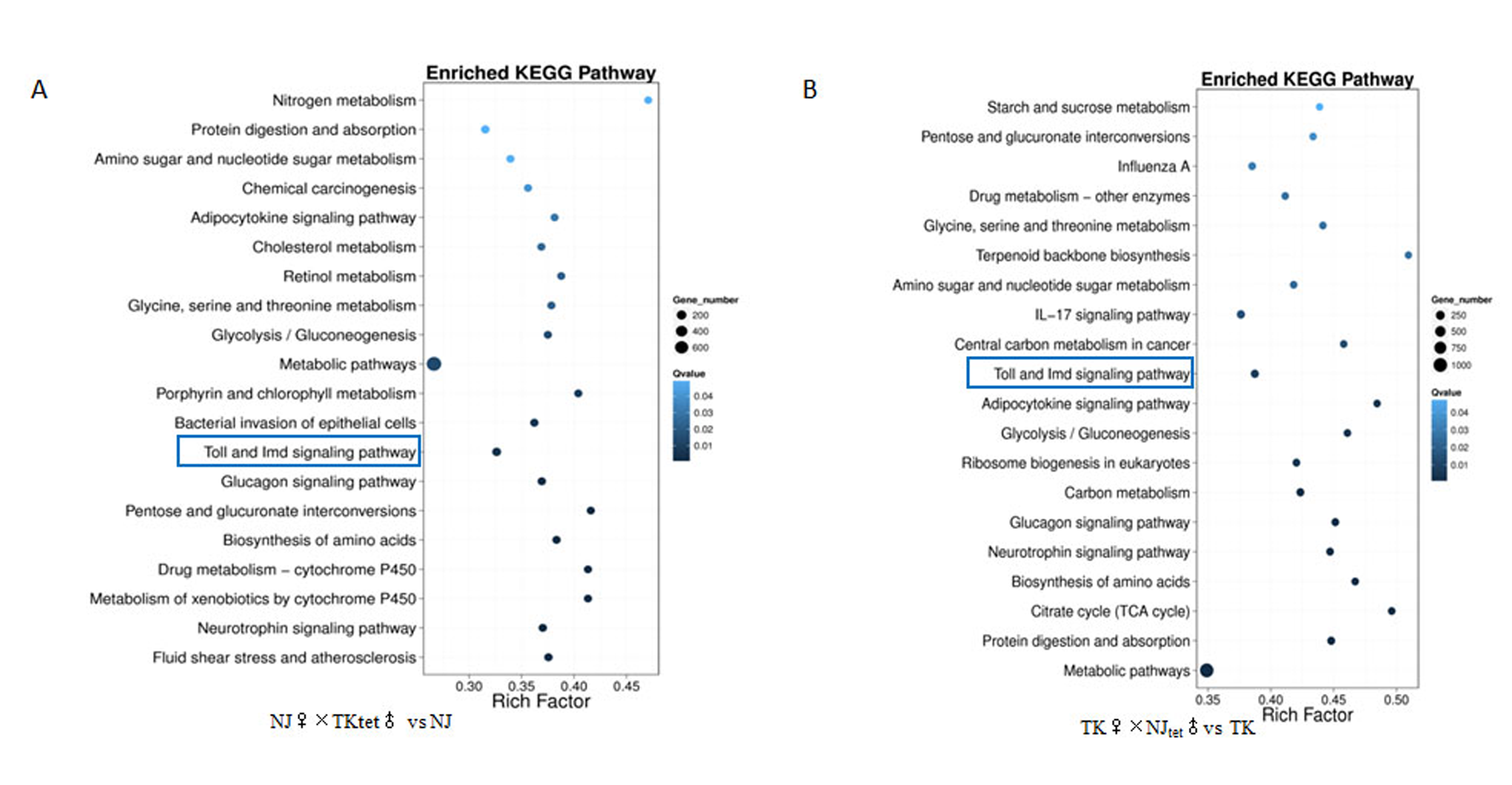

Supplement: S2 Fig — In parallel, the unigenes were mapped onto the canonical pathways in KEGG to identify possible active biological pathways of DEGs in biological replicate of RNA sequencing experiment. Twenty most significant DEGs in new vs. old host-Wolbachia symbiosis are shown here. X axis represents enrichment factor. Y axis represents pathway name. The color indicates q value (high: white, low: blue), a lower q value indicates a more significant enrichment. Point size indicates DEG number (A bigger dot refers to a larger amount). Rich Factor refers to the value of enrichment factor, which is the quotient of foreground value (the number of DEGs) and background value (total Gene amount). A larger Rich Factor value indicates a higher level of enrichment. (TIF) [file pone.0226736.s002.tif]
